# Transcriptome analysis reveals ADAMTS15 is a potential inflammation-related gene in remote ischemic postconditioning

**DOI:** 10.3389/fcvm.2023.1089151

**Published:** 2023-05-10

**Authors:** Bo Zuo, Sha Zhu, Guisong Wang, Zhengpeng Li

**Affiliations:** ^1^ Department of Cardiology, Cardiovascular Centre, Beijing Friendship Hospital, Capital Medical University, Beijing, China; ^2^ Department of Cardiology, Peking University Third Hospital, Beijing, China; ^3^ Key Laboratory of Cardiovascular Molecular Biology and Regulatory Peptides, Ministry of Health, Beijing, China; ^4^ Key Laboratory of Molecular Cardiovascular Sciences, Ministry of Education, Beijing, China; ^5^ Department of Neurology, Peking University International Hospital, Beijing, China; ^6^ Department of Gastroenterology and Hepatology, The First Medical Center, Chinese People’s Liberation Army General Hospital, Beijing, China

**Keywords:** acute myocardial infarction, ischemia reperfusion injury, inflammation, remote ischemic postconditioning (RIPostC), ADAMTS15, transcriptome analysis

## Abstract

**Background:**

Remote ischemic postconditioning (RIPostC) induced by brief episodes of the limb ischemia is a potential therapeutic strategy for myocardial ischemia/reperfusion injury, achieved by reducing cardiomyocyte death, inflammation and so on. The actual mechanisms underlying cardioprotection conferred by RIPostC remain unclear. Exploring gene expression profiles in myocardium at transcriptional level is helpful to deepen the understanding on the cardioprotective mechanisms of RIPostC. This study aims to investigate the effect of RIPostC on gene expressions in rat myocardium using transcriptome sequencing.

**Methods:**

Rat myocardium samples from the RIPostC group, the control group (myocardial ischemia/reperfusion group) and the sham group were performed transcriptome analysis using RNA sequencing. The levels of cardiac IL-1β, IL-6, IL-10 and TNFα were analyzed by Elisa. The expression levels of candidate genes were verified by qRT-PCR technique. Infarct size was measured by Evans blue and TTC staining. Apoptosis was assessed by TUNEL assays and caspase-3 levels were detected using western blotting.

**Results:**

RIPostC can markedly decrease infarct size and reduce the levels of cardiac IL-1β, IL-6 and increase the level of cardiac IL-10. This transcriptome analysis showed that 2 genes were up-regulated (Prodh1 and ADAMTS15) and 5 genes (Caspase-6, Claudin-5, Sccpdh, Robo4 and AABR07011951.1) were down-regulated in the RIPostC group. Go annotation analysis showed that Go terms mainly included cellular process, metabolic process, cell part, organelle, catalytic activity and binding. The KEGG annotation analysis of DEGs found only one pathway, amino acid metabolism, was up-regulated. The relative mRNA expression levels of ADAMTS15, Caspase-6, Claudin-5 and Prodh1 were verified by qRT-PCR, which were consistent with the RNA-seq results. In addition, the relative expression of ADAMTS15 was negatively correlated with the level of cardiac IL-1β (*r* = −0.748, *P *= 0.005) and positively correlated with the level of cardiac IL-10 (*r* = 0.698, *P *= 0.012). A negative correlation statistical trend was found between the relative expression of ADAMTS15 and the level of cardiac IL-6 (*r* = −0.545, *P *= 0.067).

**Conclusions:**

ADAMTS15 may be a potential inflammation-related gene in regulation of cardioprotection conferred by remote ischemic postconditioning and a possible therapeutic target for myocardial ischemia reperfusion injury in the future.

## Introduction

In recent years, early reperfusion therapy has become the most effective treatment for patients with acute myocardial infarction (AMI). However, as a side effect of early reperfusion treatment, ischemia reperfusion (IR) injury almost inevitably causes cardiomyocyte death or no reflows, seriously affecting the prognosis of patients with AMI (1, 2). Therefore, it is necessary to seek effective methods to alleviate IR injury at present. Remote ischemic conditioning (RIC), referring to repeated and transient ischemia/reperfusion episodes of distant organs such as limbs, could effectively reduce myocardial IR injury. In 1993, Przyklenk et al. found that remote ischemic preconditioning (RIPC), performed before prolonged myocardial ischemia, could protect the heart against myocardial IR injury (3). As the time of acute coronary occlusion is unpredictable, the application of RIPC has been greatly limited. Therefore, RIC implemented immediately following myocardial reperfusion, called remote ischemic postconditioning (RIPostC), attracts the attentions of researchers. In 2013, Crimi et al. divided 100 patients with AMI receiving primary percutaneous coronary intervention (PCI) into RIPostC group together with PCI control group, and found that the area under the CK-MB curve decreased significantly in RIPostC group (4). Furthermore, a series of clinical and basic studies confirmed the cardioprotective effect of RIPostC, which made it a feasible strategy in treatment of AMI.

At present, the mechanism of cardioprotection conferred by RIPostC is not clear (5, 6). Previous studies have shown that RIC can induce cardioprotective protein factors, such as hypoxia-inducible factor 1α (HIF-1α), stroma cell-derived factor-1 (SDF-1), nitrite, etc. These factors are usually up-regulated in heart tissue and can activate signal transduction pathways in myocardium, finally lead to significant cardioprotection (7–10). In view of current research highlights, the inflammation hypothesis of cardioprotection becomes an attractive target. RIC has been proved to play a significant anti-inflammatory role in myocardial IR injury and relate to decreased levels of the pro-inflammatory cytokines, such as interleukin-1β (IL-1β) and interleukin-6 (IL-6), after myocardial reperfusion (11). Cai et al. found RIPC can induce the increase of interleukin-10 (IL-10) which was mostly considered as a protective anti-inflammatory cytokine in plasma and heart, and then activate the protective signal pathway in the heart to induce the protection of myocardial IR injury (12–14).

In addition, previous studies on the mechanism of cardioprotection conferred by RIC are mainly focusing on the levels of protein expressions (15–17). The proteomics technology played a prominent role in revealing the mechanism of cardioprotection conferred by RIC. In 2012, Michele hepponstall et al. carried out proteomic analysis on the plasma of 5 healthy volunteers treated with limb RIPC. They found that the expression of 6 proteins changed by using 2-dimensional difference in gel electrophoresis and the expressions of 48 proteins changed by using liquid chromatography-mass spectrometry (LC-MS). The expression of apolipoprotein A-I, fibrin and fibrinogen was changed in the above two technologies (18). Further studies showed that apolipoprotein A-I up-regulated in plasma and heart tissue and played a cardioprotective role in RIPC (19). Transcriptome research is also an important method of functional genome research, which can explore gene expressions and functions more efficiently but is insufficient in RIC research (20, 21). To clarify the effects of RIPostC on gene expressions at transcriptional level in myocardium using transcriptome analysis is of great significance to figure out the mechanism of RIPostC. Therefore, this study aims to investigate the effect of RIPostC on gene expressions in rat myocardium using RNA sequencing.

## Materials and methods

### Animals

The animal experiment was approved by the Animal Care Committee of Peking University. This study was performed in accordance with the Guide for the Care and Use of Laboratory Animals published by the US National Institutes of Health (NIH Publication No. 85-23, revised 1996). Eighteen male Sprague-Dawley (SD) rats (22) with body weights of 250–300 g were used in this study and were divided into three groups randomly (*n* = 6):

RIPostC: 30 min of myocardial ischemia followed by 3 h of reperfusion. Four cycles of 5 min ischemia and 5 min reperfusion on lower limbs were applied immediately after 30 min myocardial ischemia.
Con (Control): the same treatment as RIPC, except that no ischemia on lower limbs was applied.
Sham: sham operation was implemented on rats.

### Myocardial IR model

SD rats were anesthetized and then subjected to 30 min myocardial ischemia by ligation of left anterior descending coronary artery, followed with 3 h reperfusion by releasing the ligation to simulate myocardial IR.

### The implementation of RIC

Tourniquets were used to bind both lower limbs of rats (22), causing bilateral femoral artery occlusion. After the lower limbs ischemia lasting for 5 min, the tourniquets were relaxed and the lower limbs were reperfusion for 5 min, which was repeated for 4 times. The signs of successful femoral artery occlusion were pale skin, decreased body temperature and loss of pulse. Reperfusion is marked by rapid hyperemia and redness of the distal limb, with return of body temperature and presence of pulse.

### Assessment of myocardial infarct size and inflammatory level

After myocardial reperfusion in rats, the anterior descending coronary artery was ligated at the original ligation position again. 2 ml of 1% Evans blue solution was injected to dye the non-ischemic myocardium into blue. The heart was frozen and 4–5 slices of frozen heart tissue were quickly cut. The slices were placed into 1% TTC-PBS buffer and incubated in a constant temperature water bath at 37°C for 20 min in dark. Then heart slices were transferred into 10% formalin solution. The infarction area was gray-white, the non-ischemic area was blue, and the ischemic risk area was red and white. Image J software was used for analysis and myocardial infarction area (%) was valued by infarction area/risk area. Rat troponin I (TnI) in plasma and cardiac IL-1β, IL-6, IL-10 and TNFα were analyzed by enzyme linked immunosorbent assay (Elisa) using commercial kits according to the manufacturer's protocol (Abcam, USA).

### Terminal deoxynucleotidyl transferase mediated dUTP nick end labelling (TUNEL) assay

For the detection of TUNEL-positive cells, the In Situ Cell Death Detection Kit (Roche, CH) was used according to the manufacturer's instructions.

### Western blotting

Western blotting was carried out according to the previous literature description (12). Bands were normalized to eukaryotic translation initiation factor 5 (eIF5) expression.

### Transcriptome sequencing

Rat myocardium samples from the RIPostC group (*n* = 3) and the control group (*n* = 3) were used for transcriptome analysis by RNA sequencing. Trizol (Invitrogen, USA) was used to extract total RNA from tissue samples. The concentration and purity of RNA were detected using Nanodrop2000. The integrity of RNA was measured by agarose gel electrophoresis, and the value of RNA integrity was acquired using Agilent2100. The cDNA library was established according to the instructions of Illumina TruseqTM RNA sample prep kit. The cDNA library was then sequenced using Illumina novaseq 6000.

### Quantitative reverse transcription PCR (qRT-PCR)

Total RNA was extracted from tissues using Trizol (Invitrogen, USA). A total of 2*μ*g RNA were transcribed into cDNA using PrimeScript™ RT Reagent Kit (TAKARA, Japan) according to the manufacturer's instructions. Amplification was performed using SYBR Green Real Time PCR Master Mix (TOYOBO, Japan). The sequences of primers are shown in Table 1. The relative amount of mRNA was calculated by 2^−ΔΔCT^ method. We used α-tubulin as a housekeeping gene, and all reactions were executed in triplicate for the 12 samples (*n* = 6 for RIPostC group and Con group).

**Table 1:** Primer sequences used in qRT-PCR.

Gene	Forward primer (5′-3′)	Reverse primer (5′-3′)
Caspase-6	AGCATGACGTGCCATTGGT	ACGTTGTCGTCCAGCTTGTCT
Prodh1	ACTGTGGACCTACTGGACTGGAAC	CATCCTCTTCATCTGCTGCTCTTCC
Adamts15	AAATGTGGCGTGTGTGGTGGAG	CTGGCGGATGTCAATGCTGGAG
Claudin-5	TACTCAGCACCAAGGCGAACCAC	GCGGCTTCCCACATCGGTC
α-tubulin	TTGAGCGCCCAACCTACACT	TCAGGGCCCCATCAAATCT

### Bioinformatic analysis

Raw reads were processed with fastp software (version 0.19.5). Clean reads were obtained after removal of reads containing poly-N and low-quality reads. Mapped reads were acquired by comparison of clean reads with reference genome Rattus_Norvegicus (Rnor_6.0) using hisat software (version 2.1.0). Then mapped reads were assembled using StringTie (version 2.1.2) software.

The genes/transcripts were compared with NR (version 2020.06), GO (version 2020.0628) and KEGG (version 2020.07) databases, and the annotation of each database was statistically analyzed. TPM (transcripts per million reads) was obtained by RSEM (version 1.3.3) software to detect the expression levels of genes and transcripts. Venn analysis was performed to evaluate the common and unique expressed genes/transcripts. Principal Component Analysis (PCA) was carried out to access the relationship and variation between samples using R (version 3.3.1) software package. DESeq2 (Version 1.24.0) software was used to identify differentially expressed genes (DEGs). An adjusted *P-*value <0.05 and fold change >2 or fold change <0.5 were considered as thresholds for significantly differential expressions (23). The volcano map and heatmap for DEGs was conducted by R (version 3.3.1). GO and KEGG annotation for DEGs was completed using R (version 3.3.1) software package.

### Statistical analysis

The data are expressed in mean ± standard error (SE). R (version 3.3.1) software and Graphpad Prism 8.0 (La Jolla, CA, USA) were used for statistical analysis. Variables were tested for normal distribution using the Kolmogorov–Smirnov test and Q–Q plots. Student's *t* test was used to compare variables with normal distribution and the Mann–Whitney *U* test was used to compare variables without a normal distribution. *P *< 0.05 was considered to be statistically significant. Pearson's correlation was used to investigate the association between the relative expression of ADAMTS15 (2^−ΔCT^ method) and cardiac inflammatory indicators.

## Results

### RIPostC induce protection against myocardial IR injury in rat model

**Figure 1:**
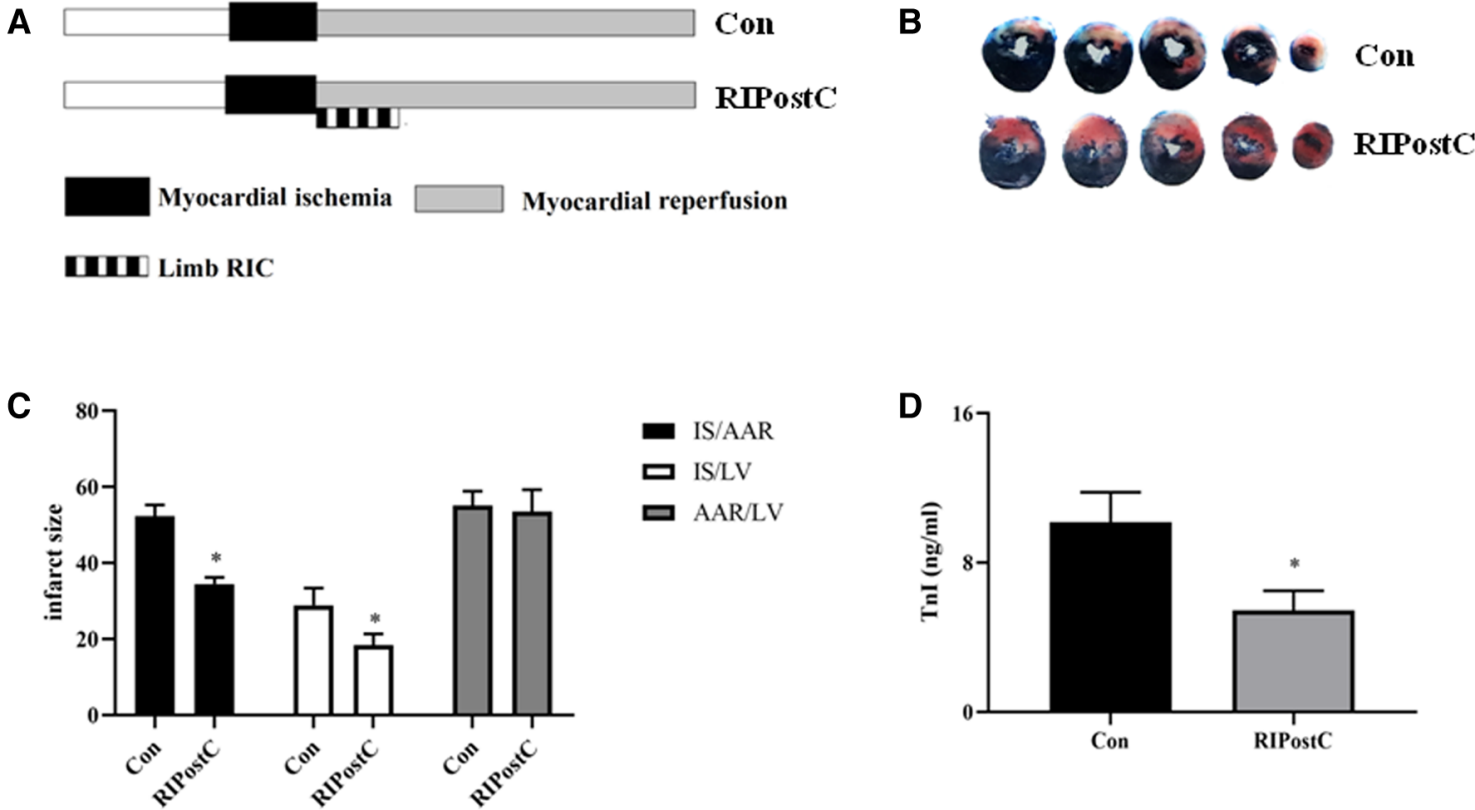
RIPostC significantly reduced cardiac injury in rat myocardial IR model. (**A**) The protocol of experimental design. (**B,C**) Evans blue and TTC staining. Representative five heart slices stained by Evans blue and TTC in two groups. IS, infarct size; AAR, the ischemic area at risk; LV, left ventricle. (**D**) The level of plasma TnI. *Represents *P* < 0.05, *n* = 6 for each group.

**Figure 2:**
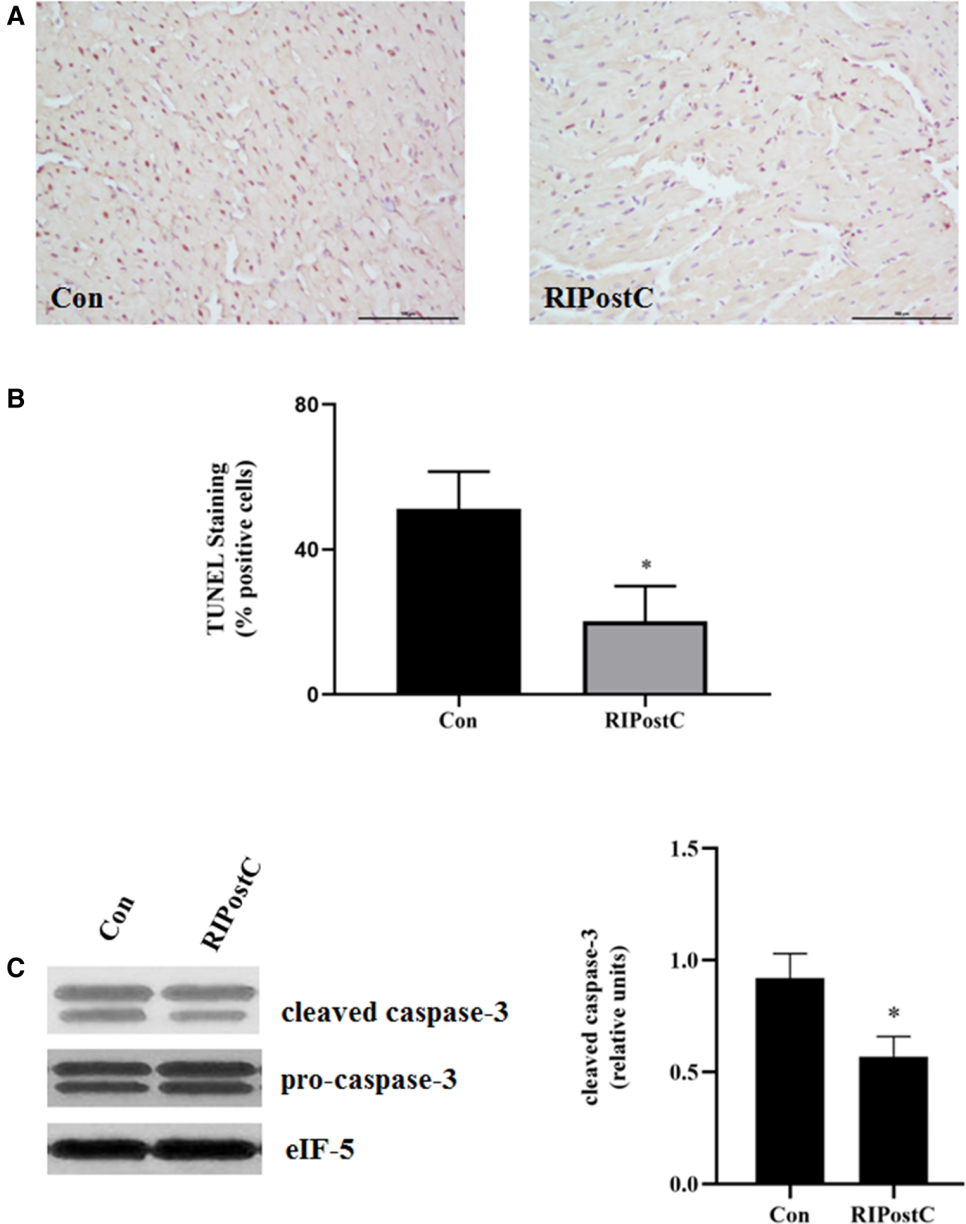
RIPostC significantly reduced the apoptosis of heart. (**A**) Representative myocardial apoptosis detected by TUNEL assay. (**B**) The percentage of TUNEL-positive cells in the total cells. Scale bar: 100 µm. *Represents *P* < 0.05, *n* = 6 for each group. (**C**) Detection of caspase-3 levels in heart after RIPostC using western blotting.

Rats were subjected to RIPostC immediately after prolonged myocardial ischemia (Figure 1A). We assessed the cardioprotective effect of RIPostC by detecting myocardial infarct area in myocardial IR model using Evans blue-TTC staining method. RIPostC markedly reduced infarct size compared with the Con group (Figures 1B,C). Besides, the level of plasma TnI significantly decreased in the RIPostC group (Figure 1D). In addition, compared to the Con group, the number of TUNEL-positive cells significantly decreased in the RIPostC group, and the level of cleaved-caspase 3 significantly downregulated in the RIPostC group, showing that RIPostC could markedly suppress cardiac apoptosis during myocardial IR (Figure 2 and Supplementary Figure S1). These data illustrated that RIPostC can confer powerful protection against myocardial IR injury.

### RIPostC significantly reduce cardiac inflammation in myocardial IR injury

**Figure 3:**
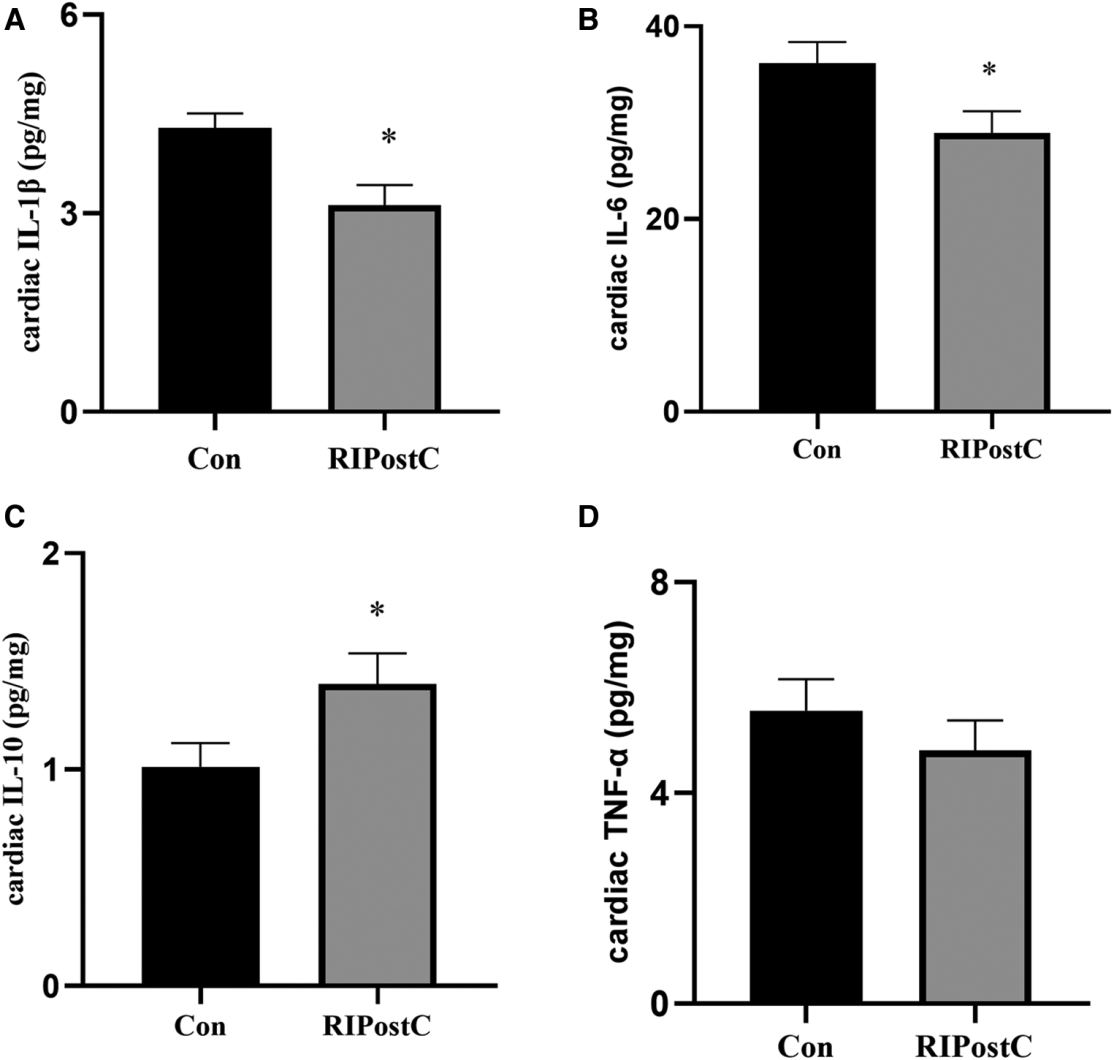
The comparison of the levels of cardiac inflammatory factors between the Con and the RIPostC group. (**A–D**) The comparison of cardiac IL-1β, IL-6, IL-10 and TNFα levels respectively in the RIPostC and the Con group. *Represents *P* < 0.05, *n* = 6 for each group.

Compared to the Con group, the levels of cardiac IL-1β and IL-6 significantly decreased in the RIPostC group and the level of cardiac IL-10 significantly elevated (Figure 3 and Supplementary Figure S2). These data showed that RIPostC can play a powerful anti-inflammatory role in myocardial IR injury.

### Expression differences between the Con and the RIPostC group

**Figure 4:**
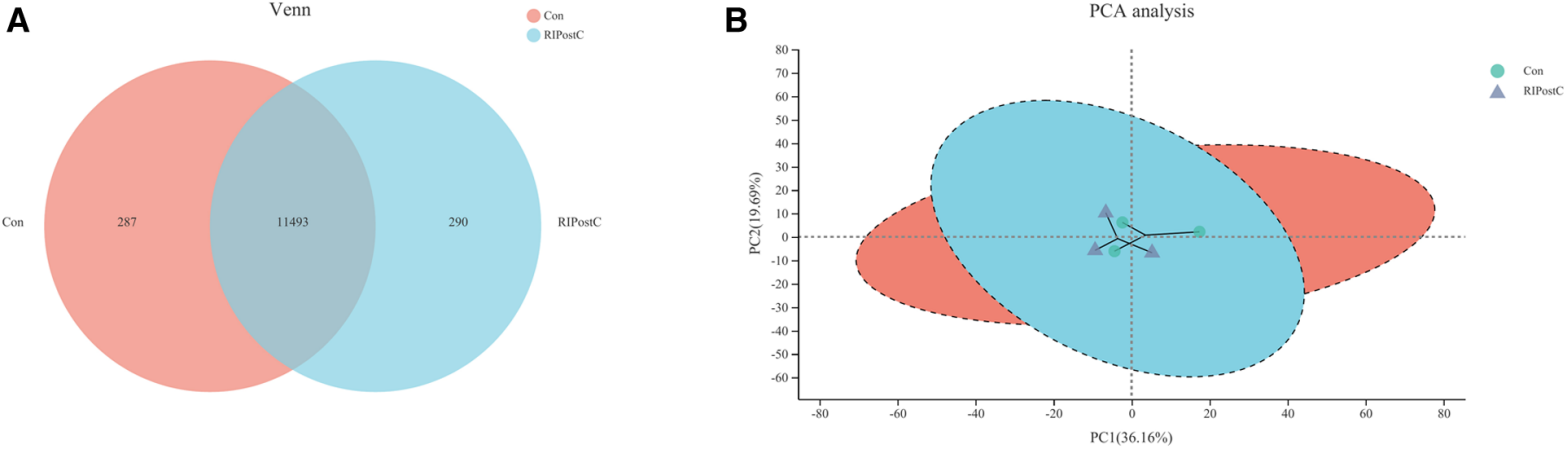
Expression differences between the Con and the RIPostC group. (**A**) Shared and unique expressed genes displayed by Venn analysis. (**B**) Differences of expression pattern exhibited by PCA analysis.

The transcriptome analysis of 6 samples was completed, the clean reads of each sample was more than 7.26 GB, and the percentage of Q30 base was >94.38%. The clean reads of each sample were mapped with the specified reference genome, and the alignment rate was >95.29%. 11,493 known genes were expressed in the Con group as well as the RIPostC group, while 287 and 290 genes were only expressed in the Con group or the RIPostC group respectively (Figure 4A). PCA results showed that differences existed in a certain extent on gene expression patterns between the two groups (Figure 4B).

### DEGs identification and analysis

**Figure 5:**
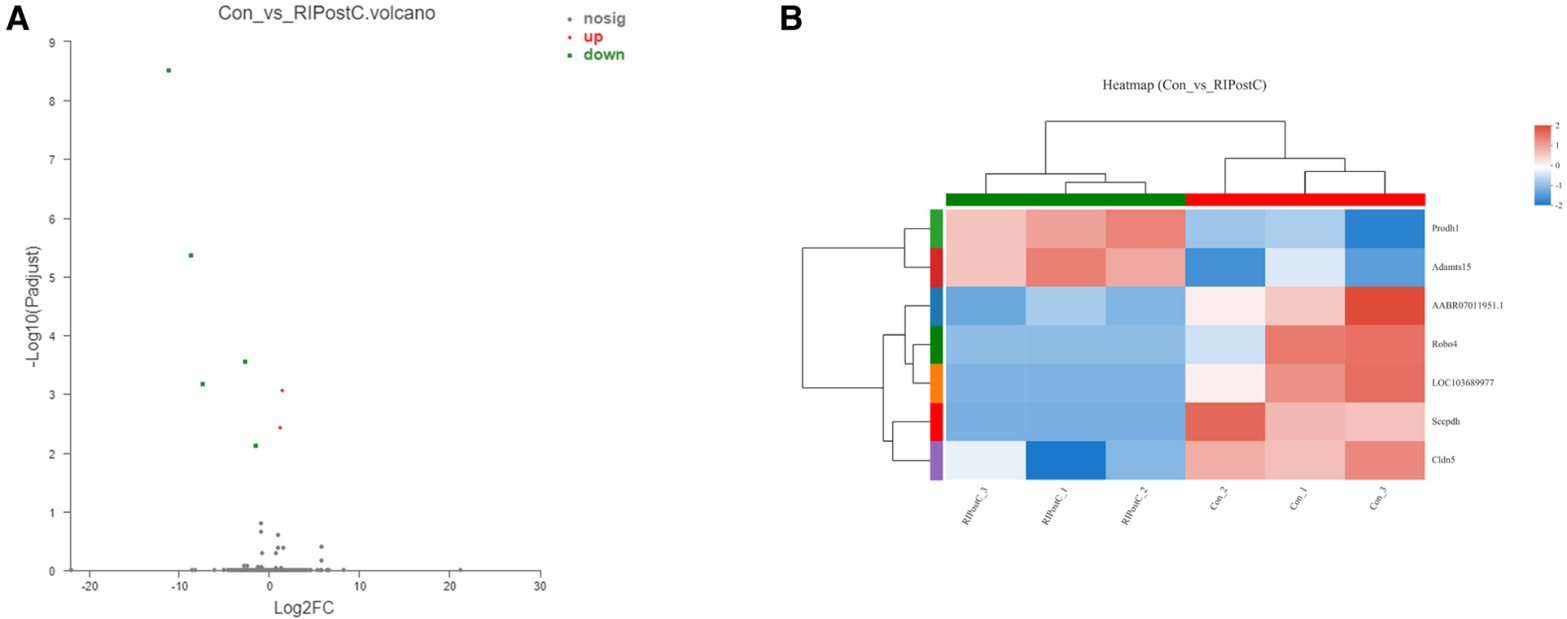
DEGs identification and analysis. (**A**) Red and green dots represented up and down regulated genes by Volcano map. (**B**) The expression levels of DEGs and similarity between samples as well as DEGs showed by heatmap.

Volcano map showed that the number of DEGs in the RIPostC group vs. to the Con group was 7. The heatmap displayed the expression levels of DEGs in every sample between two groups. The 2 up-regulated genes are: ADAMTS15 (ADAM metallopeptidase with thrombospondin type 1 motif, 15) and Prodh1 (proline dehydrogenase 1). The 5 down-regulated genes are: Cldn5 (Claudin-5), Sccpdh (saccharopine dehydrogenase), Robo4 (roundabout guidance receptor 4), LOC103689977 (Caspase-6) and AABR07011951.1 (Heat shock cognate 71 kDa protein) (Figure 5). Expression differences between the Con and the Sham group were shown in Supplementary Figure S3 and Table S1.

### GO and KEGG annotation analysis of DEGs

**Figure 6:**
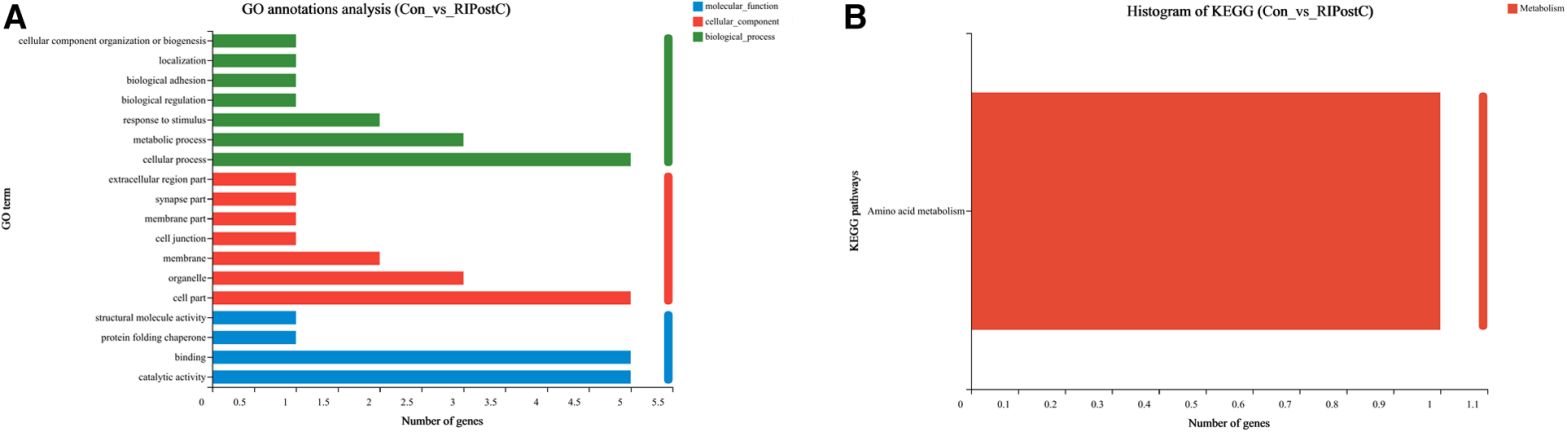
Go and KEGG annotation analysis of DEGs. (**A**) Annotated GO terms of DEGs. (**B**) Annotated KEGG pathways of DEGs.

To identify the functions of DEGs induced by RIPostC, GO terms were annotated. In biological process, cellular process, metabolic process and response to stimulus correspond to 5, 3 and 2 DEGs respectively. In cellular component, cell part, organelle, and membrane correspond to 5, 3 and 2 DEGs respectively. In molecular function, catalytic activity and binding correspond to 5 different genes respectively. Therefore, this GO annotation analysis showed that GO terms mainly included cellular process, cell part, binding, catalytic activity, organelle and metabolic process (Figure 6A). Meanwhile, the KEGG annotation analysis of DEGs between the two groups found that only one pathway, amino acid metabolism, was up-regulated (Figure 6B).

### Verification of RNA sequencing data by qRT-PCR

**Figure 7:**
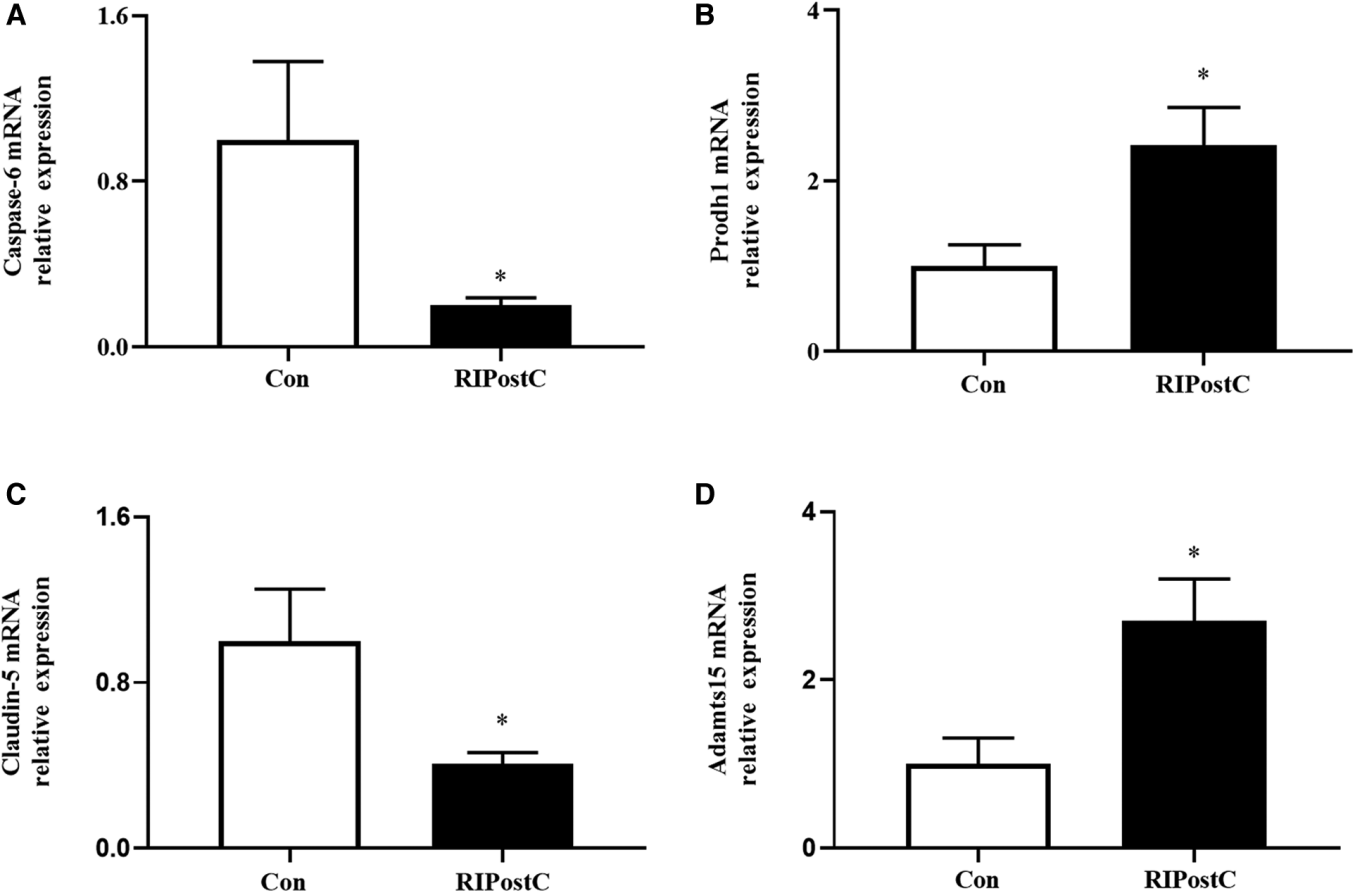
Expression levels of DEGs validated by qRT-PCR. (**A–D**) The relative mRNA expression comparisons of Caspase-6, Prodh1, Claudin-5 and ADAMTS15 respectively in the RIPostC and the Con group. *Represents *P *< 0.05, *n* = 6 for each group.

The expression levels of candidate genes were verified by qRT-PCR. As shown in Figure 7, compared to Con group, the relative mRNA expression levels of Caspase-6 and Claudin-5 were decreased, Prodh1 and ADAMTS15 were increased in the RIPostC group, which were consistent with the RNA-seq results.

### The correlation between the relative expression of ADAMTS15 and cardiac inflammatory indicators

**Figure 8:**
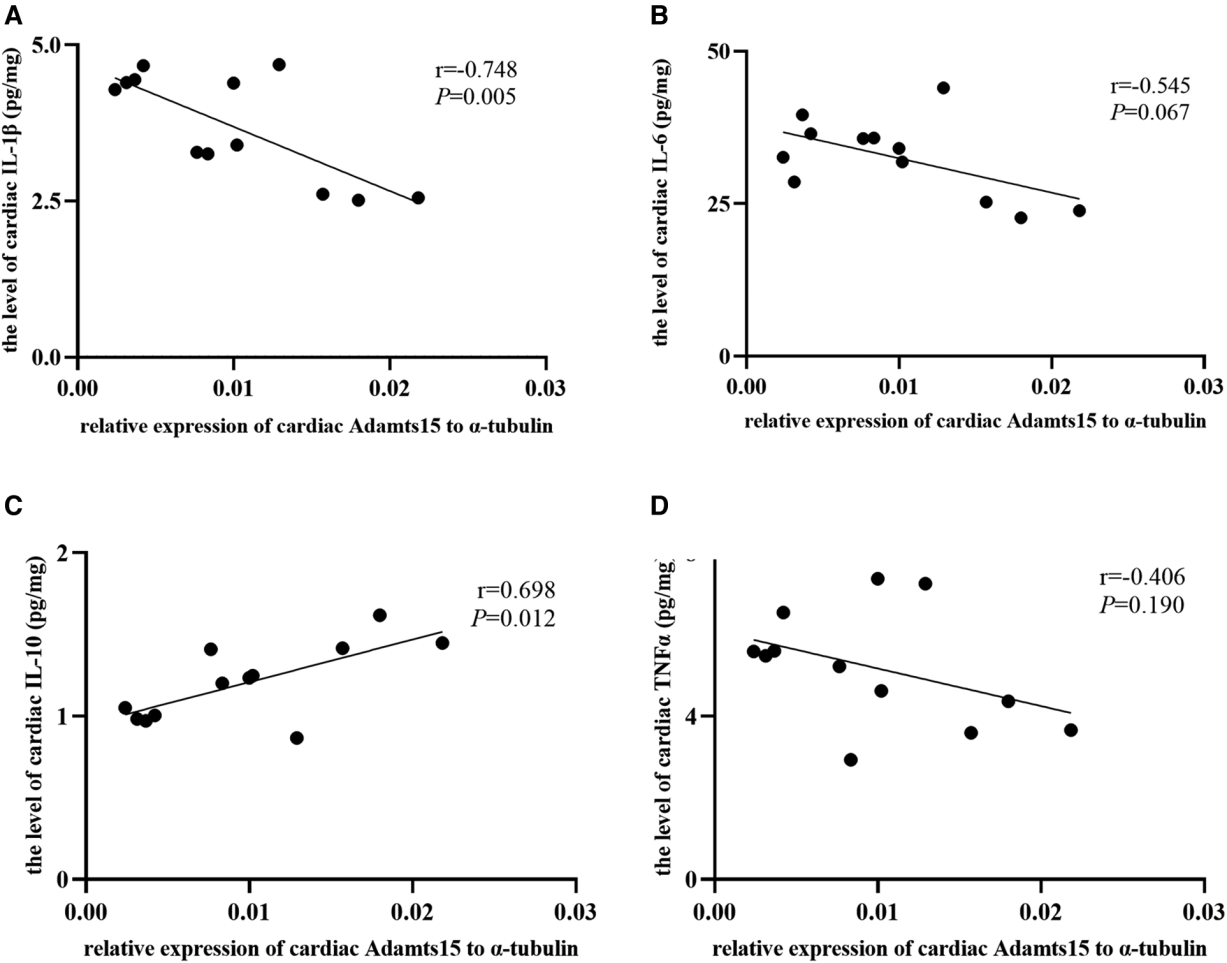
The correlation between the relative expression of ADAMTS15 and the levels of cardiac inflammatory factors. (**A–D**) The correlation between the relative mRNA expression of ADAMTS15 and the levels of cardiac IL-1β, IL-6, IL-10 and TNFα respectively in the RIPostC and the Con group. *Represents *P* < 0.05, *n* = 6 for each group.

The scatter diagrams and correlation analysis demonstrated that the relative expression of ADAMTS15 were corelated to the levels of cardiac inflammatory indicators (Figure 8). The relative expression of ADAMTS15 was negatively correlated with the level of cardiac IL-1β (*r* = −0.748, *P *= 0.005) and positively correlated with the level of cardiac IL-10 (*r* = 0.698, *P *= 0.012). In addition, a negative correlation trend was found between the relative expression of ADAMTS15 and the level of cardiac IL-6 (*r* = −0.545, *P *= 0.067). There was no statistical correlation between the relative expression of ADAMTS15 and the level of cardiac TNF-α (*r* = −0.406, *P *= 0.190).

## Discussion

Inflammation is a key factor of myocardial IR injury. Excessive inflammation mediates a series of interactions between neutrophils and vascular endothelial cells, which plays a fundamental role in the inflammatory process of myocardial IR injury. Inflammatory signal cascades triggered during myocardial IR injury can lead to the release of proinflammatory cytokines, such as IL-1β, IL-6 and IL-18, promote leukocyte adhesion to endothelial cells, and trigger leukocyte infiltration into inflammatory damage. Therefore, anti-inflammatory strategy is a potential effective therapeutic method to alleviate myocardial IR injury (11, 24). In several studies, RIC was associated with the reduction of proinflammatory cytokines, such as IL-1β, IL-6 (11). Besides, RIC was proved to increase the level of protective cytokine IL-10. The up-expression of cardiac IL-10 can alleviate the cytokine response and lead to significant cardioprotection in RIC (12). Consistent with the results in previous studies, we found that RIPostC can reduce the levels of cardiac IL-1β, IL-6 and increase the level of cardiac IL-10, showing powerful anti-inflammatory role of RIPostC.

In this study, we investigated the effect of RIPostC on gene expressions in myocardium by using transcriptome sequencing in rat models. Our results showed that 7 genes were differently expressed between the RIPostC group and the Con group. Go analysis showed that DEGs associated GO terms mainly included cellular process, cell part, binding, catalytic activity, organelle and metabolic process. Further KEGG annotation analysis showed that these DEGs are associated with amino acid metabolism pathway. Compared to the Con group, the relative mRNA expression levels of Caspase-6 and Claudin-5 decreased, Prodh1 and ADAMTS15 increased in the RIPostC group by qRT-PCR, which were consistent with the RNA-seq data. In addition, the relative expression of ADAMTS15 was negatively correlated with the level of cardiac IL-1β and positively correlated with the level of cardiac IL-10. To our knowledge, this is the first study to investigate the transcriptome changes in the myocardium using RNA sequencing technology and demonstrate the overall mechanism changes induced by RIPostC. The potential role of ADAMTS15 in myocardial IR injury and RIPostC was a novel discovery.

ADAMTS are members of proteolytic enzyme family, which are secreted by fibroblasts, smooth muscle cells, macrophages and other cells (25). ADAMTS is a kind of secreted zinc endopeptidase, which contains a signal peptide, a variable length anterior domain, a metalloproteinase domain, an integrin like domain, a central platelet reactive protein type 1 sequence repeat (TSR) motif, a cysteine rich spacer domain and auxiliary domain. The auxiliary domain determines the differences among members of the ADAMTS protein family. ADAMTS are associated with a variety of diseases, including cardiovascular disease (25). Clinical studies have shown that low ADAMTS13 level was associated with an increased risk of AMI (26). ADAMTS5 is considered to have a protective effect on atherosclerosis by regulating the catabolism of vascular proteoglycan and improving the deposition of lipoprotein (27). The expression level of ADAMTS7 was up-regulated in damaged plaques and may promote vascular smooth muscle cells migration and proliferation and aggravate vascular remodeling (28). Therefore, ADAMTS can play key roles in the occurrence and development of coronary heart disease.

Interestingly, ADAMTS is a family of enzymes closely related to acute and chronic inflammation. It is increasingly recognized that inflammation stimulates thrombosis, which in turn promotes inflammation. Up to now, ADAMTS were proved to be key contributors to thrombo-inflammation. For example, literatures have shown that ADAMTS13 gene deficiency mice have proinflammatory and thrombogenic phenotypes. In addition, the administration of recombinant ADAMTS13 can alleviate inflammation in myocardial IR injury. ADAMTS13 can prevent excessive platelet and/or leukocyte recruitment in ischemic myocardium by cleaving von Willebrand factor (vWF). The treatment of wild type mice with recombinant human ADAMTS13 resulted in a smaller infarct area and decreased number of neutrophils infiltrating ischemic myocardium, indicating that ADAMTS13 had an effective anti-inflammatory effect during myocardial IR injury (29, 30).

Similar with other ADAMTS members, ADAMTS15 is also a metalloproteinase with thrombospondin domain. It was reported that ADAMTS15 played a tumor suppressor role in prostate cancer and colorectal cancer (31). However, to our knowledge, the role of ADAMTS15 in myocardial IR injury and RIPostC has not been investigated. In this study, we found that the relative expression of ADAMTS15 was negatively correlated with the level of cardiac IL-1β and positively correlated with the level of cardiac IL-10, indicating that ADAMTS15 may be a potential inflammation-related gene in cardioprotection conferred by RIPostC and a possible effective target for myocardial IR injury. The inflammation-related role and mechanism of ADAMTS15 in RIC need to be further studied in future.

In this study, we found that the expression of Caspase-6 was down-regulated after the implementation of RIPostC. Caspases is a family of enzymes that regulate programmed cell death, inflammation and other biological functions. Caspase-6 is generally identified as an executioner of apoptosis, a kind of non-inflammatory cell death (32, 33). In contrast to apoptosis, other common programmed cell death forms, pyroptosis and necroptosis, for instance, may result in the increase of interleukins release, such as IL-1β and IL-18, to enhance cardiac inflammation in myocardial IR injury, mostly considered to be associated with activation of NLRP3 inflammasome and NF-κβ pathway. Recent studies showed that Caspase-6 may facilitate ZBP1-mediated inflammasome activation and promote the activation of programmed cell death pathways, including pyroptosis and necroptosis (34). Whether Caspase-6, with or not with ADAMTS15, involves in cardioprotection conferred by RIC via regulating inflammation related pyroptosis and necroptosis is an interesting hypothesis and worth exploring in the future.

Claudin-5 is a transmembrane protein, which is widely expressed in epithelium and endothelial cells of brain, heart and so on. Claudin-5 was found to mediate the occurrence and development of cerebral ischemia-reperfusion injury (35, 36). In the ischemic penumbra, the level of Claudin-5 decreased in the early stage of vascular remodeling and increased in the later stage (37). However, the change of Claudin-5 expression over time in myocardial IR was still unknown. The role and mechanism of Claudin-5 in RIPostC need to be further investigated in future. In this study, the expression of Prodh1 is up-regulated after RIPostC. Prodh1 is a kind of enzyme that catalyzes the first step of proline catabolism (38). Wang et al. found that the enhancement of proline metabolism induced by over-expression of prodh decreased the level of reactive oxidative stress and apoptosis, while prodh gene knockout had the opposite effect in rat cardiomyocytes under hypoxia, indicating a cardioprotective role by the enhancement of proline metabolism (39).

Our KEGG annotation analysis showed that amino acid metabolism pathway may involve in regulation of cardioprotection conferred by RIPostC. In 2016, a metabolomic study showed that RIPC was associated with a decrease in ornithine and an increase in kynurenine (KYN) together with glycine concentrations in both rat and human plasma samples (40). Further study showed that inhibition of KYN synthesis may eliminate the cardioprotective effect induced by RIPC (41). At present, there are few studies on the role of amino acid metabolism in cardioprotection induced by RIC, which needs further exploration in our future research work.

Meanwhile, there are some limitations in this study. First, our results of transcriptome sequencing are based on a small sample size, which may not provide enough information to support our hypothesis. Second, the subjects of this study were rats, and species heterogeneity may lead to differences in results (42). Third, RIC is a physiological process involving multiple targets, such as limb, heart and blood. This study only focused on the expressions of DEGs in myocardial tissue and could not fully reflect the mechanism of RIC. Finally, as an association study, this research lacks evidence for causality between the transcriptomics changes and the observed cardioprotection. Thus, further study should be performed to explore how these DEGs involve in cardioprotection induced by RIC, especially ADAMTS15. In addition, the mechanism of the cardioloprotective role of RIPostC should be explored by ex-vivo experiments in the future.

## Conclusion

In conclusion, 7 differently expressed genes were found after RIPostC by using transcriptome sequencing in rat models. ADAMTS15 may be a potential inflammation-related gene in regulation of cardioprotection conferred by RIPostC and a possible therapeutic target for myocardial IR injury in future. Caspase-6 mediated cell death may possibly involve in cardioprotection induced by RIPostC. Amino acid metabolism pathway, may involve in regulation of cardioprotection. Further studies are needed to explore the complex mechanisms of cardioprotection induced by RIPostC.

## Data Availability

The data presented in the study were deposited in the NCBI repository which can be found below: https://www.ncbi.nlm.nih.gov/bioproject/PRJNA847371/.
