# Supplementary material for: Transcriptome analysis reveals ADAMTS15 is a potential inflammation-related gene in remote ischemic postconditioning
Source: Front Cardiovasc Med. 2023 May 10;10:1089151. doi: 10.3389/fcvm.2023.1089151 (PMC10206167; doi:10.3389/fcvm.2023.1089151)
Supplement: Supplementary file 2 [file Datasheet1.docx]

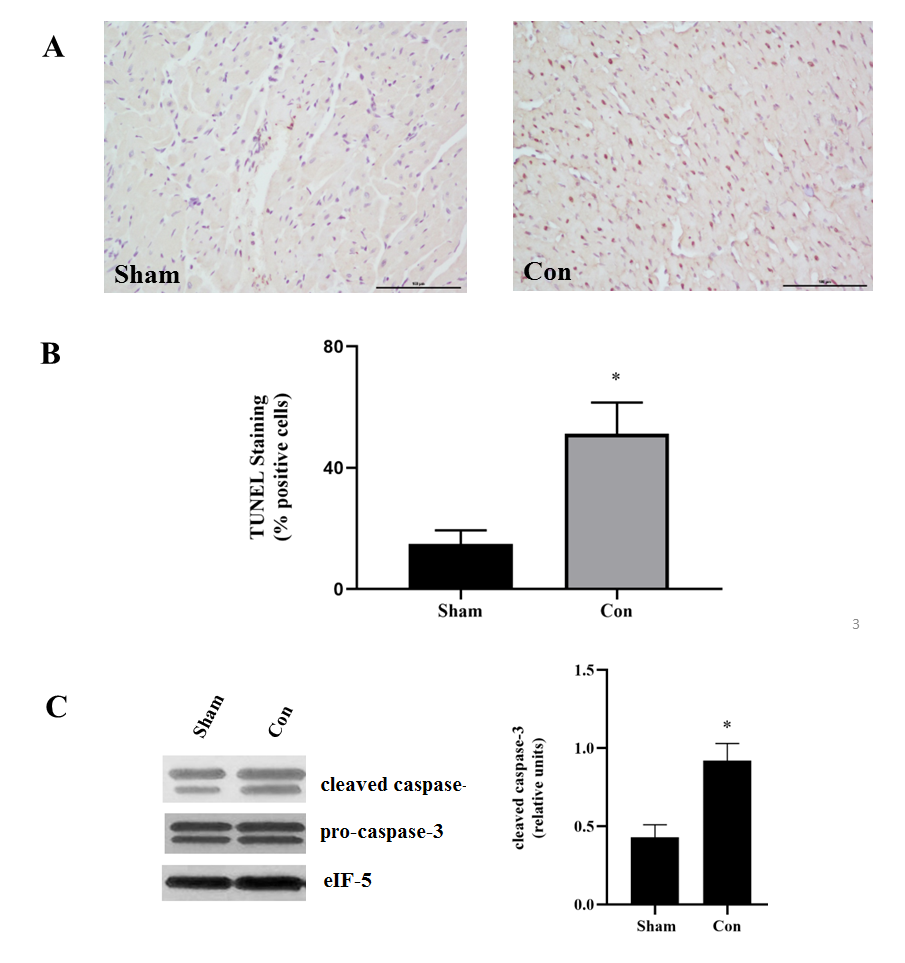


**Figure S1 RIPostC significantly reduced** t**he apoptosis of heart between the Con and the Sham group**. **A**: Representative myocardial apoptosis detected by TUNEL assay. **B**: The percentage of TUNEL-positive cells in the total cells. Scale bar: 100 μm. * represents P< 0.05, n=6 for each group. **C**: Detection of caspase-3 levels in heart using western blotting.


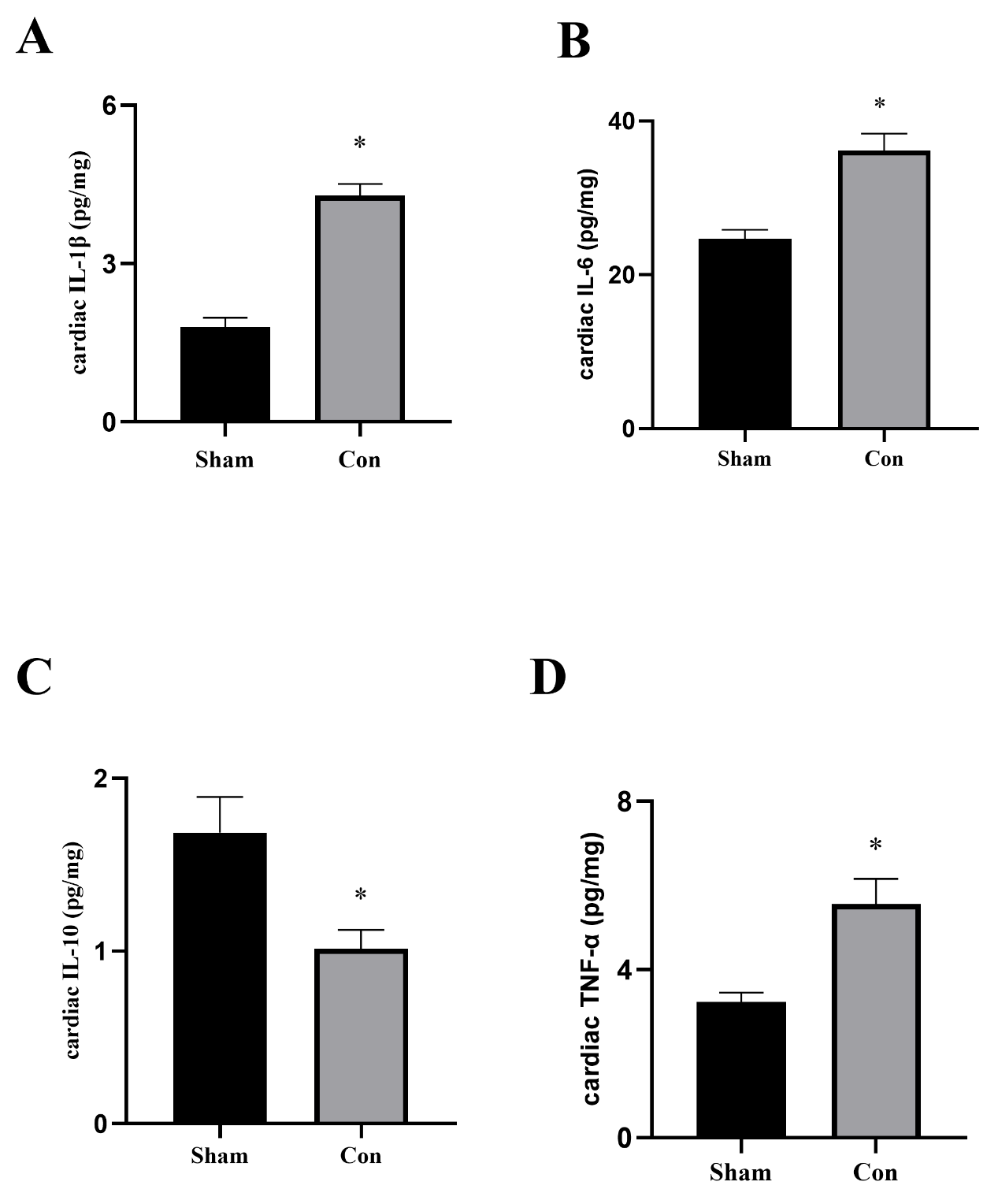


**Figure S2 The comparison of the levels of cardiac inflammatory factors between the Con and the Sham group**. **A-D**: The comparison of cardiac IL-1β, IL-6, IL-10 and TNFα levels respectively in the Con and the Sham group. * represents P<0.05, n=6 for each group.


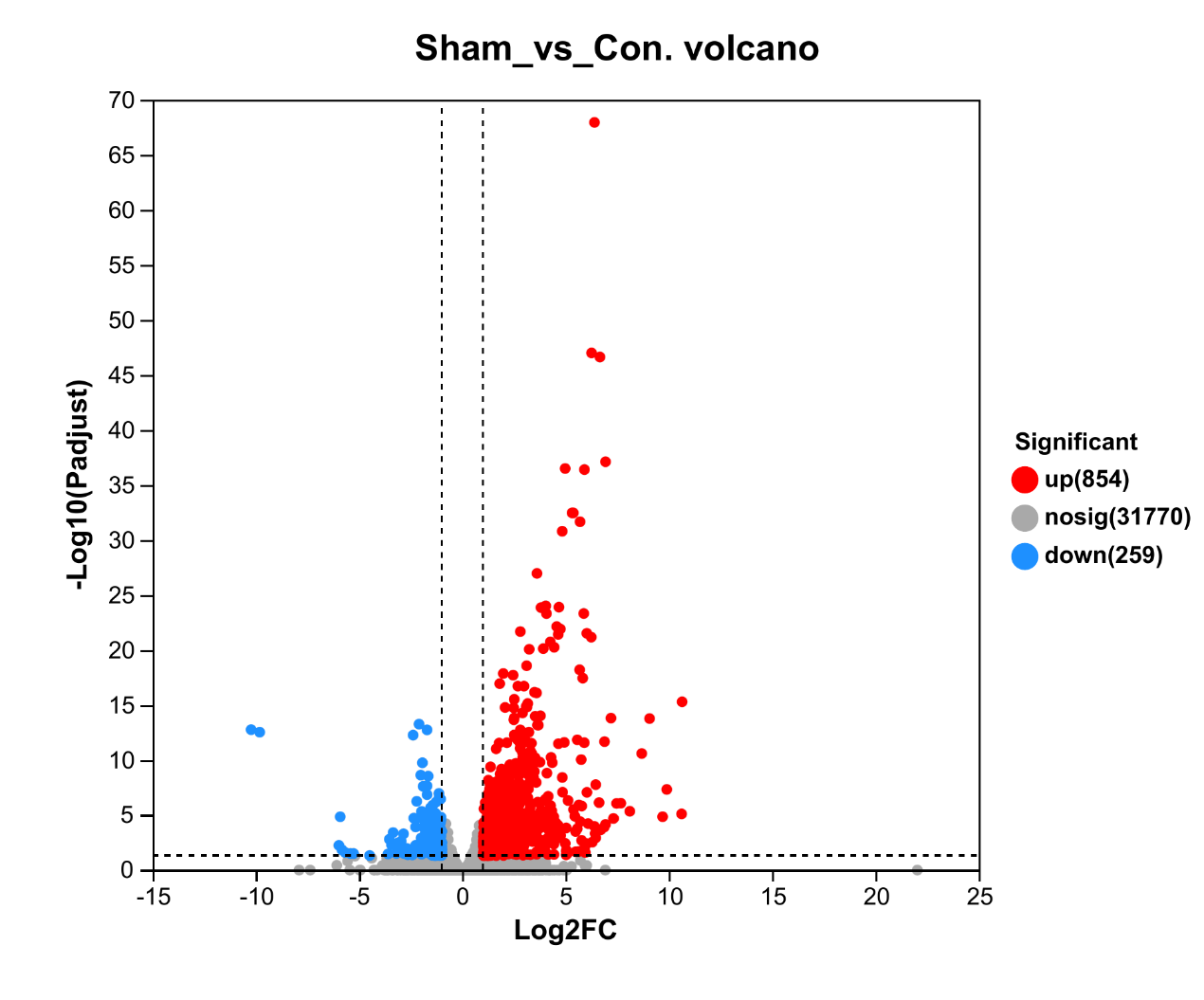


**Figure S3 DEGs identification by Volcano map between the Con and Sham group**. Red and blue dots represented up and down regulated genes.

**Table S1 The expression levels of DEGs between the Con and the Sham group.**

Refer to the table of supplementary material.
